# Semiautomated segmentation of breast tumor on automatic breast ultrasound image using a large-scale model with customized modules

**DOI:** 10.1038/s41598-025-97098-w

**Published:** 2025-05-19

**Authors:** Yi Zhou, Mingtao Ye, Haiyang Ye, Shuqi Zeng, Xi Shu, Ying Pan, Aifen Wu, Pengpeng Liu, Guodao Zhang, Shibin Cai, Shuzheng Chen

**Affiliations:** 1 Department of Breast Surgery, The Fifth Affliated Hospital of Wenzhou Medical University, LiShui Municipal Central Hospital, Lishui, 323000 China; 2 Institute of Intelligent Media Computing, Hangzhou Dianzi University, Hangzhou, 310018 China; 3 Department of Ultrasound, The Fifth Affliated Hospital of Wenzhou Medical University, LiShui Municipal Central Hospital, Lishui, 323000 China; 4 Department of Breast Surgery, Lishui Maternal and Child Health Care Hospital, Lishui, 323050 China

**Keywords:** Ultrasonography, Breast neoplasms, Deep learning, Breast cancer, Breast cancer, Computational models

## Abstract

To verify the capability of the Segment Anything Model for medical images in 3D (SAM-Med3D), tailored with low-rank adaptation (LoRA) strategies, in segmenting breast tumors in Automated Breast Ultrasound (ABUS) images. This retrospective study collected data from 329 patients diagnosed with breast cancer (average age 54 years). The dataset was randomly divided into training (n = 204), validation (n = 29), and test sets (n = 59). Two experienced radiologists manually annotated the regions of interest of each sample in the dataset, which served as ground truth for training and evaluating the SAM-Med3D model with additional customized modules. For semi-automatic tumor segmentation, points were randomly sampled within the lesion areas to simulate the radiologists’ clicks in real-world scenarios. The segmentation performance was evaluated using the Dice coefficient. A total of 492 cases (200 from the “Tumor Detection, Segmentation, and Classification Challenge on Automated 3D Breast Ultrasound (TDSC-ABUS) 2023 challenge”) were subjected to semi-automatic segmentation inference. The average Dice Similariy Coefficient (DSC) scores for the training, validation, and test sets of the Lishui dataset were 0.75, 0.78, and 0.75, respectively. The Breast Imaging Reporting and Data System (BI-RADS) categories of all samples range from BI-RADS 3 to 6, yielding an average DSC coefficient between 0.73 and 0.77. By categorizing the samples (lesion volumes ranging from 1.64 to 100.03 cm^3^) based on lesion size, the average DSC falls between 0.72 and 0.77.And the overall average DSC for the TDSC-ABUS 2023 challenge dataset was 0.79, with the test set achieving a sora-of-art scores of 0.79. The SAM-Med3D model with additional customized modules demonstrates good performance in semi-automatic 3D ABUS breast cancer tumor segmentation, indicating its feasibility for application in computer-aided diagnosis systems.

## Introduction

Breast cancer, as one of the most common cancers among women, has a global incidence rate that continues to rise, profoundly impacting women’s health^1^. The importance of early breast cancer screening lies in enhancing the likelihood of early detection, enabling prompt treatment, thereby improving the cure and survival rates, and alleviating patient suffering. Common screening methods for breast cancer include mammography and two-dimensional handheld ultrasound (HHUS). Mammography has limited sensitivity in detecting dense breast tissue and involves radiation exposure. Additionally, mammography may apply pressure to the breasts during the imaging process, leading to discomfort for women with sensitive breasts^2^. HHUS is relatively limited in obtaining three-dimensional breast structural information and may be difficult to accurately depict the location and shape of lesions. Additionally, a subjective examination method may lead to poor reproducibility among different doctors^3,4^.

To enhance breast cancer screening more effectively, several advanced breast ultrasound technologies and devices have emerged in recent years, with a significant advancement being three-dimensional automated breast ultrasound (ABUS) technology^5^. ABUS utilizes rotational sensors and three-dimensional scanning to obtain high-quality images of the entire breast. In contrast, ABUS provides more comprehensive breast images without radiation exposure, and its high-resolution images and comprehensive coverage enhance the detection of breast lesions with greater sensitivity and accuracy. However, a three-dimensional ABUS image typically comprises hundreds of slices, making the review of all ABUS images a time-consuming task, even for experienced radiologists^6^. And doctors might be dependent on ABUS imaging^7^. Computer-aided diagnosis (CAD) systems, designed to assist doctors with complex and large-scale image diagnostics, can help radiologists and clinicians analyze tumor characteristics and develop surgical plans^8,9^, for which accurate automatic/semi-automatic ABUS lesion segmentation is a prerequisite for the feasibility of CAD systems. However, due to indistinct lesion boundaries, significant class imbalance, and noise from imaging, segmenting lesions in ABUS images remains a challenge^10^.

In recent years, the field of medical image segmentation has witnessed significant advances, particularly the widespread use of deep learning algorithms, which have greatly improved the segmentation accuracy of medical images^11–13^. Various architectures, including convolutional neural networks (CNNs)^14–16^, transformer-based models^17^, and hybrid approaches^18,19^, have been proposed to tackle the challenges posed by the complex anatomical structures and heterogeneous appearance of breast lesions in ABUS images. Despite these advances, challenges such as high inter-patient variability and limited annotated datasets hinder the robustness and generalization of existing methods. To address these limitations, large-scale models pre-trained on diverse datasets have emerged as a promising solution. Moreover, large models such as GPT-4^20^, SAM^21^, and SAM-Med3D^22^ are trained on massive datasets, allowing them to broadly address requirements across various domains. Implementing these large models offers significant benefits in the product field, where a single model can cater to the needs of multiple specific tasks. However, these large-scale computer vision (CV) models often face challenges in being directly applied to specific medical tasks due to a lack of medical image data training. Fine-tuning large models to cater to specific domain needs has emerged as a current research focus. Full fine-tuning, as one of the most common fine-tuning techniques, enhances the performance of large models in specific downstream domains by further training them on datasets, yet this approach of updating all parameters is often constrained by the high costs associated with the model’s extensive parameters.

In summary, this study explores the use of a low-rank fine-tuning strategy^23,24^ to customize one of the representative CV large models, SAM-Med3D, for semi-automatic segmentation of breast cancer tumors in ABUS. The performance of the model in applying 3D ABUS semi-automatic breast cancer tumor segmentation was evaluated across multiple datasets.

## Material and methods

### Patient

**Fig. 1:**
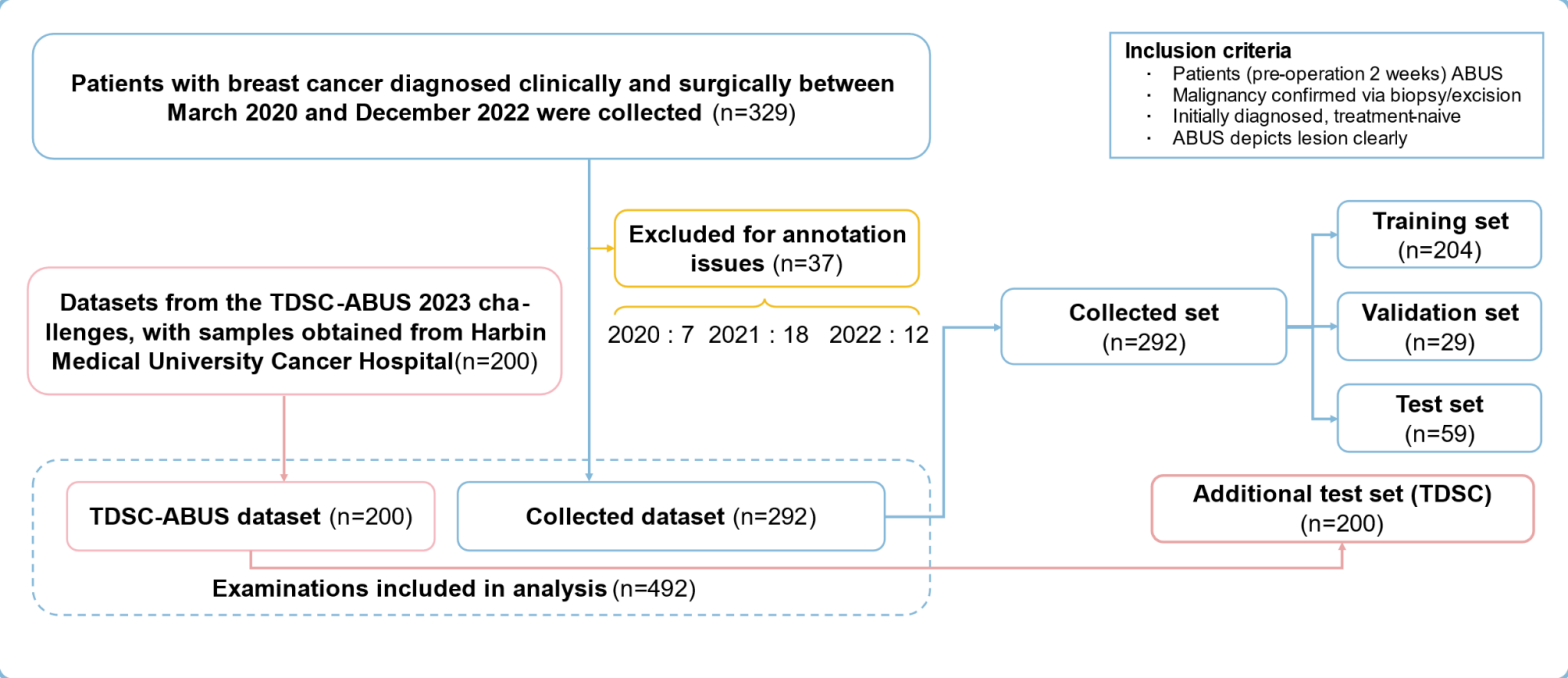
This figure illustrates the dataset composition used in our study, including the distribution of training, validation, and test sets. It also highlights the inclusion of the external TDSC-ABUS dataset as an independent test set, clearly distinguishing it from the data collected at our institution.

This retrospective single-center study was approved by the Ethics Review Committee of Lishui Central Hospital, including but not limited to data collection and use. We confirmed that all methods were performed in accordance with the relevant guidelines and regulations. Due to the retrospective nature of the study, Ethics Review Committee of Lishui Central Hospital waived the need of obtaining informed consent. The study collected 329 patients with breast cancer diagnosed clinically and pathologically from Lishui Central Hospital from March 2020 to December 2022, as shown in Fig. 1. The inclusion criteria for patients are as follows: (1) All lesions were confirmed as malignant tumors by core needle puncture or surgical resection; (2) All patients underwent ABUS examination within 2 weeks before puncture or surgery; (3) All patients were diagnosed with breast cancer for the first time and had not received any treatment; (4) The extent of the lesion could be clearly identified on the ABUS image. The exclusion criteria were as follows: (1) Poor quality ABUS images or non-nodular lesions; (2) Patients who had previously undergone breast implantation or breast deformity, which are not suitable for ABUS examination; (3) Patients who had received any form of anti-cancer treatment (including surgery, chemoradiotherapy, endocrine therapy, etc.) before ABUS examination; (4) Patients with malignant tumors in other parts of the body. Additionally, an external test dataset containing 200 patients with meticulous tumor labels was obtained from the TDSC-ABUS challenge^25^. The automatic three-dimensional breast ultrasound images of the patients were acquired by the ABUS device (Invenia™ Automated Breast Ultrasound System, GE Healthcare, USA). Among them, the system version used by the Lishui Central Hospital was 1.0, and the TDSC-ABUS challenge was 2.0.

Of the 292 patients collected and confirmed by our hospital, 204 cases were used as the training set, 29 cases were used as the validation set, and 59 cases were used as the test set. Additionally, 200 datasets collected by Harbin Medical University for the TDSC-ABUS 2023 challenge were included as an extra test set. The model’s generalization performance in unfamiliar organizational environments was assessed across these two test sets.

### ABUS examination

Using the Automated Breast Volume Scanner (GE Healthcare, America), ultrasound coupling gel was evenly applied to one breast. A disposable coupling membrane was placed over the scanner. The operator positioned the scanner on the breast and gently pressed it to ensure contact with the skin. Once the scanner was in place, the operator began scanning, repeating the process three times per breast for the AP, LAT, and MED quadrants to obtain a complete view of the entire breast. Additional views were taken as needed. The same method was then used to examine the other breast. After scanning, the data were transferred to the workstation.

### Ground truth for lesion segmentation

The included patient image data were visualized using an open-source software ITK-SNAP^26^. The lesions of patients’ ABUS images were delineated by two sonologists with over 10 years of experience each (Reader 1 has 8 years of experience, Reader 2 has 10 years of experience) based on the Breast Imaging Reporting and Data System (BI-RADS) to ensure accurate lesion delineation. Each reader assessed half of the cohort and reviewed each other’s delineations. Controversial samples were resolved through joint consultation between the two sonologists to eliminate discrepancies. Each patient underwent examinations from three views for each breast: Anterior–Posterior (AP), Medial–Lateral (MED), and Lateral-Transverse (AT). The sonologists selected the images with the best visibility for each patient and manually delineated the tumors using the polygon mode in ITK-SNAP. The readers fully utilized biopsy results and other examination data during the delineation process to ensure accuracy and completeness.

### Image pre-processing

The ABUS image sequences were extracted from the Invenia ABUS system and initially screened manually, resulting in data from 292 patients. For all patient data, an open-source SimpleITK library was used to convert the data to NIFTI format. Further preprocessing involved cropping and padding the images to the specified dimensions of 840 × 440 × 330. For dimensions smaller than the required size, padding was performed proportionally with zero data in both positive and negative directions. Resampling was configured to set the spatial voxel spacing to 0.2 mm, 0.082 mm, and 1 mm in the three respective directions. During the data cleaning process, patient privacy was ensured, eliminating the need for informed consent and protecting patient rights.

### Semiautomated segmentation simulation

The model simulates the radiologist’s clicking operation within the tumor by specifying random coordinate positions (x, y, z) within the corresponding slice of the tumor. For 3D medical images of lesions with a size of [H, W, D] and annotated by sonologists on D axial slices, the model performs a 3D convolution using a kernel size of (16 × 16 × 16). These features are then paired with learnable 3D absolute position encodings and fed into the 3D attention modules along with customized fine-tuning modules to learn spatial details within the 3D medical images. Simultaneously, the prompt encoder integrates the radiologist’s click information (x, y, z) with the 3D mask decoder, iteratively training and generating corresponding volume segmentations. Further simulated clicks on the unmarked lesion areas of the model’s output results guide the model in refining the segmentation, aiming to annotate the lesion areas as completely as possible.

### The architecture of SAM-Med3D with customized module

**Fig. 2:**
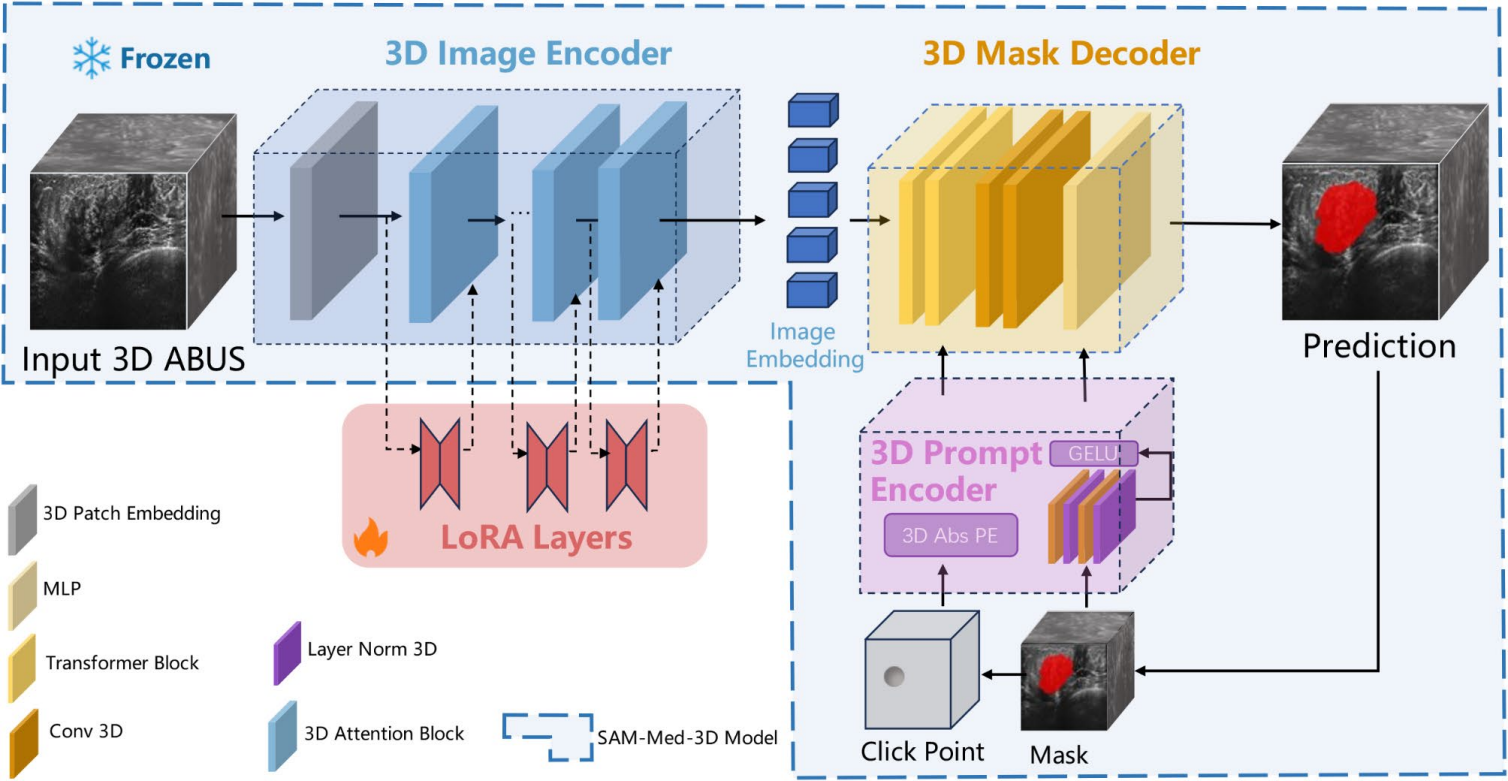
The overall structure of the SAM-Med3D model with customized module.

The structure of the proposed model with customized modules is shown in Fig. 2, which can be divided into four core components: 3D Image Encoder, 3D Mask Decoder, 3D Prompt Encoder, and LoRA Layers. In the 3D Image Encoder, 3D convolution with a kernel size of (16, 16, 16) is first used to embed image patches, complemented by learnable 3D absolute position encoding (PE) then fed into the 3D self-attention blocks, where 3D relative position encoding is introduced to capture spatial details. In the 3D Mask Decoder block, a lightweight structure efficiently maps image embeddings, accompanied by a set of prompt embeddings, to an output mask. The 3D Prompt Encoder module handles both sparse (points, boxes) and dense (masks) prompts. Sparse prompts use 3D position encoding to represent subtle 3D spatial nuances, while dense prompts are processed through 3D convolution and then combined with learned embeddings specific to each prompt type. This design enables the model to fully leverage the 3D structure to directly capture spatial information.

**Fig. 3:**
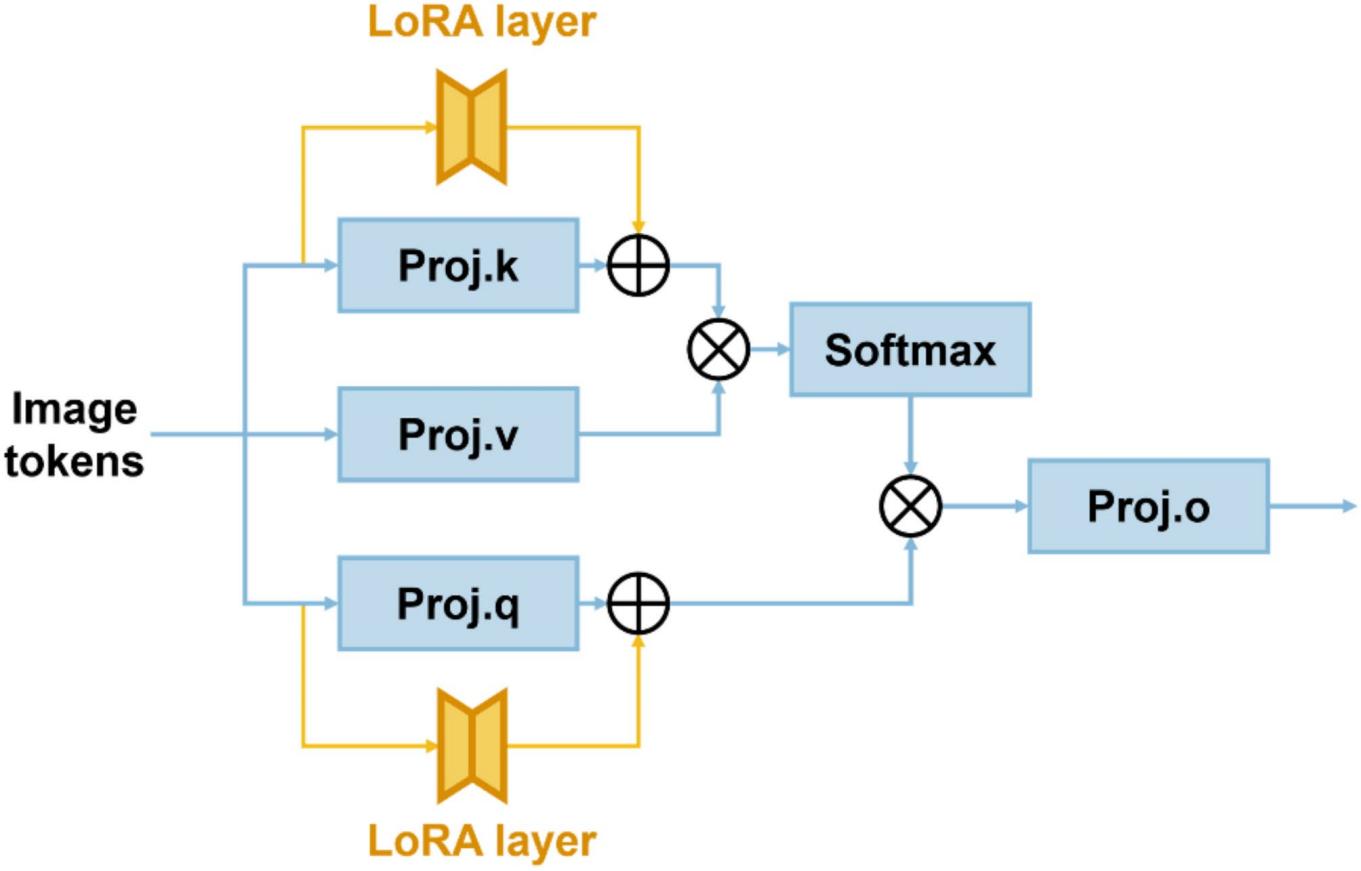
LoRA layout in the 3D Attention module.

The “LoRA Layers” is a customized module designed to adapt the model for specific segmentation tasks^24^. Since LoRA allows a large model to update only a small portion of parameters during training, it not only reduces computational overhead but also lowers the deployment and storage complexity of the fine-tuned model while maintaining segmentation performance. Each LoRA block within the LoRA Layer is attached as a trainable bypass to every 3D attention block of the frozen “3D Image Encoder”. These bypasses first compress the input features into a low-rank space, with the rank size customizable and set to 4 in this case, then perform feature re-projection to align with the output feature channels of the frozen 3D self-attention blocks, as shown in Fig. 3. The resulting customized module occupies only 29.56 MB of storage space, which is approximately 2.6% of SAM-Med3D’s size.

### Training procedure

The training process was conducted using a single NVIDIA GeForce RTX 4090 GPU, with a batch size set to 2. For each data sample, a 128 × 128 × 128 block was uniformly cropped or padded from the lesion area and fed into the model for training. During the inference and training processes, we employ multiple simulated clicks to mimic the corrective actions that a radiologist might take to refine the model’s misclassified regions, specifically false positives (FP) and false negatives (FN). The number of simulated clicks is set to 11. In each click operation, the FP and FN regions are first computed based on the ground truth and model predictions, and their union is taken (in real-world clinical scenarios, professional radiologists can identify these regions without the need for ground truth). Subsequently, a random point is selected from each 2D slice within the 3D region, and these points are combined to form a 3D set of corrective prompts. Finally, the combined prompts are fed into the prompt encoder to further enhance the segmentation performance.

To improve model robustness and generalization, a five-fold cross-validation strategy was employed. The train and validation dataset was mixed and randomly split into five subsets, with each fold serving as the validation set once while the remaining four folds were used for training. This approach ensures that every data sample is included in the validation set exactly once, reducing bias and improving the reliability of the performance evaluation. The final model performance is reported as the mean and standard deviation of the results obtained across the five folds. The combination of Cross-entropy loss and Dice loss was chosen as the cost function for the training process. Cross-entropy loss focuses on per-pixel classification accuracy, while Dice loss provides a better assessment of global segmentation quality and class balance. The combination of these two loss functions offers a more comprehensive evaluation of the segmentation model’s performance. The loss and Dice values were calculated every 20 batches. The model weights were updated using the AdamW optimizer with an initial learning rate of lr = 0.0002. For learning rate scheduling, the learning rate was adjusted at specified intervals, decreasing by a factor of 10 at the 120th and 180th epochs. The model was trained for a total of 200 epochs. The validation strategy involved validation every 10 epochs, with a batch size of 1 for validation. For validation data samples, N blocks of size 128 × 128 × 128 are cropped from the entire lesion area, where N is determined by the number of blocks required to cover the entire lesion. Blocks with lesion pixels less than 2000 (less than 0.2% of the total number of pixels in the entire block) are discarded. The model validated each lesion block individually, and the results were then stitched together based on positional information and embedded into an empty annotation file of the same size as the sample data. The Dice value and Hausdorff Distance (HD) were was calculated by comparing the predicted annotation file with the ground truth annotation file.

### Statistical analysis

The accuracy of semi-automatic segmentation is evaluated by calculating the DSC, using sonologists’ annotations as the ground truth. The DSC is a set of similarity measure function commonly used to assess the spatial overlap between the prediction results and the ground truth. Additionally, the HD is incorporated as a supplementary metric to evaluate segmentation performance. The HD measures the maximum distance from a point in one set to the closest point in another set, providing insight into the worst-case segmentation error. The analysis is divided into (a) the breast lesion grades determined by the Breast Imaging Reporting and Data System (BI-RADS) Assessment Category proposed by the American College of Radiology^27^ (with all samples in the dataset including BI-RADS 3, BI-RADS 4A, BI-RADS 4B, BI-RADS 4C, BI-RADS 5, BI-RADS 6), and (b) the measured size of the breast tumors (with tumor sizes categorized by volume into 0 ~ 5 cm^3^, 5 ~ 20 cm^3^, and 20 ~ 100 cm^3^). The breast tumor evaluation corresponding to the BI-RADS assessment levels is shown in Table 1^27,28^.

**Table 1:** BI-RADS assessment categories with the assessment result.

BI-RADS assessment categories	Assessment result
BI-RADS 0	Incomplete
BI-RADS 1	Negative
BI-RADS 2	Benign
BI-RADS 3	Probably benign
BI-RADS 4	Suspicious
BI-RADS 4A	Low suspicion of malignancy, about > 2% to ≤ 10% likelihood of malignancy
BI-RADS 4B	Intermediate suspicion of malignancy, about > 10% to ≤ 50% likelihood of malignancy
BI-RADS 4C	Moderate concern, but not classic for malignancy, about > 50% to < 95% likelihood of malignancy
BI-RADS 5	Highly suggestive of malignancy
BI-RADS 6	Known biopsy-proven malignancy

## Results

### Study population

**Fig. 4:**
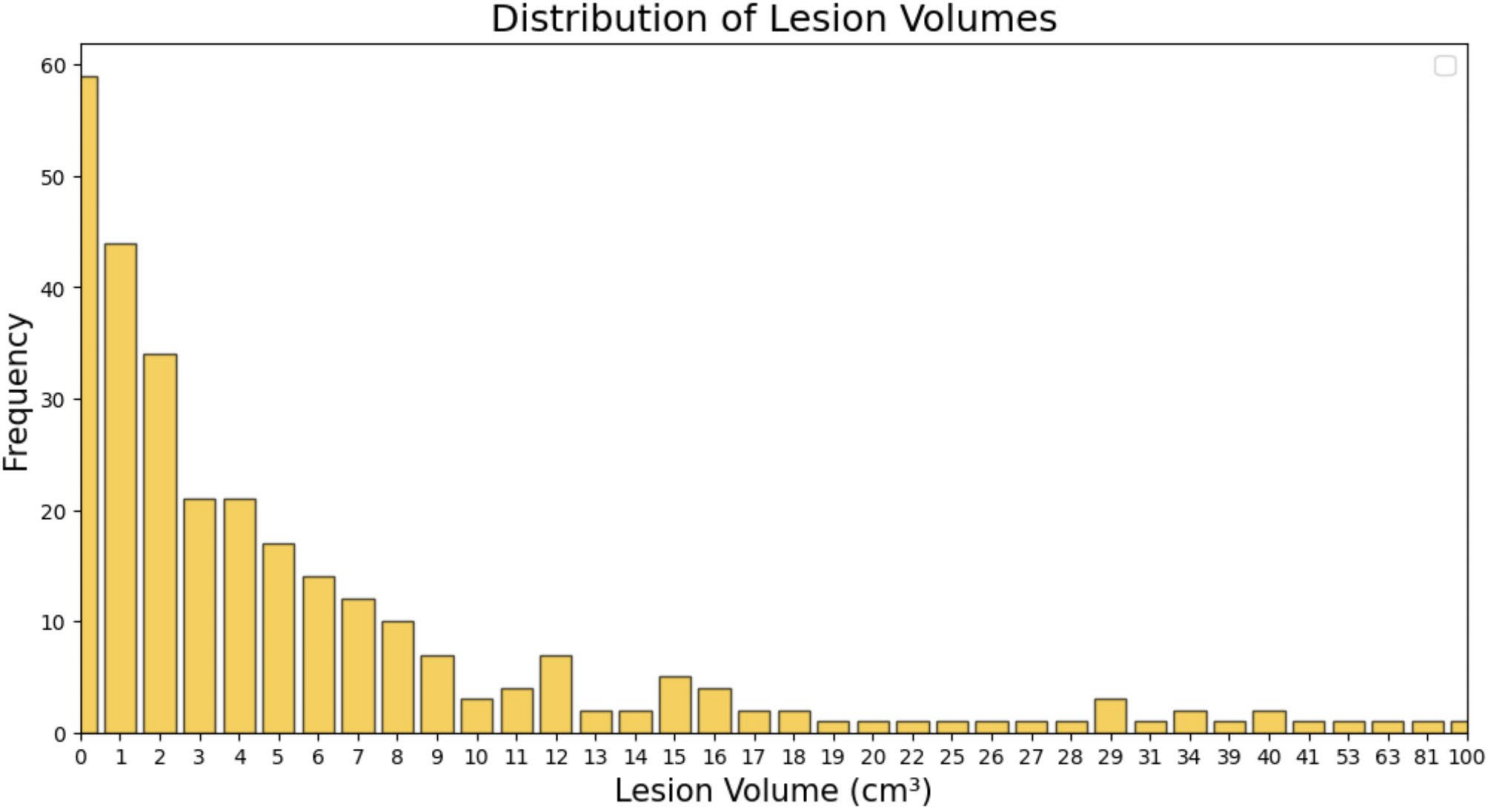
Data distribution of lesion volumes in the collected dataset.

An analysis was conducted on the collected dataset of 292 patients, with a median lesion volume of 3.39 cm^3^ and a range of 1.64 to 100.03 cm^3^. A summary of the lesion characteristics of the 292 patients from the dataset and each group of data is shown in Table 2. The distribution of lesion volumes in the dataset is illustrated in Fig. 4. A comparative analysis of the distribution of lesion volumes between the relevant collected datasets and the TDSC-ABUS 2023 dataset is shown in Table 3.

**Table 2:** Patient and lesion characteristics in the collected dataset.

	Total cohort (n = 292)	Training set (n = 204)	Validation set (n = 29)	Test set (n = 59)
Patient characteristics				
AGE (years)	27 ~ 84	27 ~ 84	35 ~ 71	31 ~ 81
BI-RADS category				
BI-RADS 3	25 (8.6%)	16 (7.8%)	3 (10.4%)	6 (10.1%)
BI-RADS 4A	36 (12.3%)	26 (12.7%)	4 (13.8%)	6 (10.1%)
BI-RADS 4B	55 (18.8%)	37 (18.1%)	7 (24.1%)	11 (18.6%)
BI-RADS 4C	124 (42.5)	89 (43.6%)	10 (34.5%)	25 (42.4%)
BI-RADS 5	31 (10.6%)	18 (8.8%)	4 (13.8%)	9 (15.3%)
BI-RADS 6	21 (7.2%)	18 (8.8%)	1 (3.4%)	2 (3.4%)
Volume of lesion				
0–5 cm3	180 (61.6%)	123 (60.3%)	19 (65.5%)	38 (64.4%)
5–20 cm3	92 (31.5%)	64 (31.4%)	9 (31.0%)	19 (32.2%)
20–100 cm3	20 (6.8%)	17 (8.3%)	1 (5.3%)	2 (3.4%)

‘%’ indicates the proportion of each category relative to the entire set.

**Table 3:** Comparative analysis of lesion volume distributions between the collected dataset and the TDSC-ABUS 2023 dataset.

Dataset	Mean ± SD (cm^3^)	Median (cm^3^)	Cohen’s d (Effect Size)	T-test *p*-value
TDSC-ABUS 2023	2.77 ± 3.27	1.49	-0.39	5.55 × 10⁻⁶
Collected dataset	6.99 ± 11.23	3.41		

### Performance of modified SAM-Med3D with customized module

**Fig. 5:**
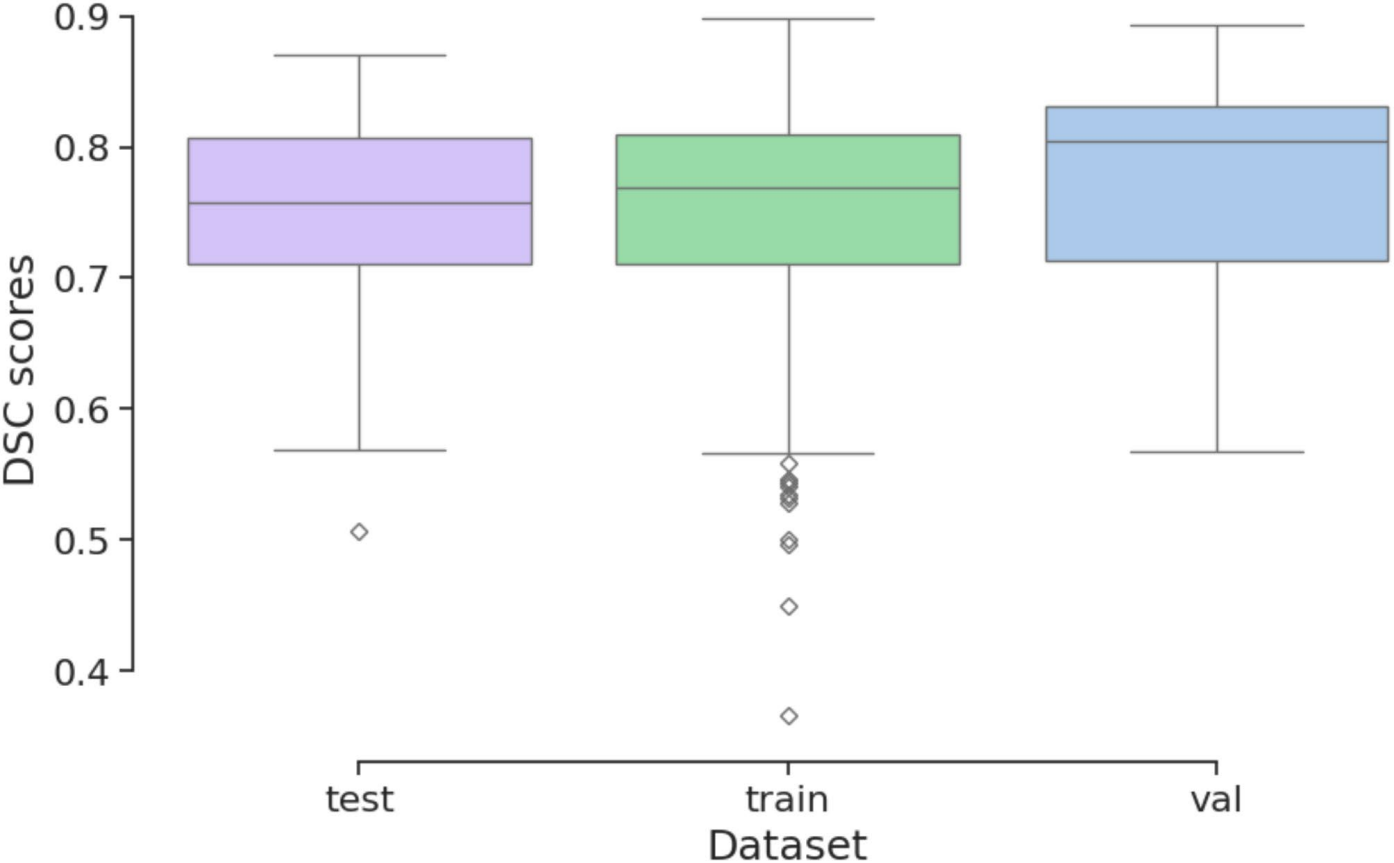
Box-plot of interference result for all samples.

The modified SAM-Med3D model with the customized module was trained, achieving DSC scores in the training set ranging from 0.37 to 0.90 (mean of 0.75), in the validation set from 0.57 to 0.89 (mean of 0.78), and in the test set from 0.51 to 0.87 (mean of 0.75). The statistical results are illustrated in Fig. 5.

Further inference was conducted using the customized module on the training and validation datasets, with results presented in Tables 4 and 5. The distribution of sample sizes and lesion BI-RADS grades in the test set were similar to those in the training and validation sets. The test dataset was manually annotated by experienced radiologists to provide ground truth data. Each sample was treated as an independent case, comparing the inference results of the SAM-Med3D model with the customized module against the radiologists’ annotations. The experimental statistics are shown in Tables 4 and 5. The average inference DICE score on the test set was 0.75. The mean HD for the test set is 60.67.

**Table 4:** DSC obtained from five-fold cross-validation inference results across all datasets, categorized by BI-RADS levels and lesion volume.

	Total cohort (n = 292)	Training set (n = 204)	Validation set (n = 29)	Test set (n = 59)
All lesions	0.75 ± 0.09	0.75 ± 0.09	0.78 ± 0.07	0.75 ± 0.08
BI-RADS category				
BI-RADS 3	0.77 ± 0.1	0.76 ± 0.12	0.79 ± 0.07	0.79 ± 0.03
BI-RADS 4A	0.75 ± 0.09	0.77 ± 0.07	0.73 ± 0.12	0.68 ± 0.1
BI-RADS 4B	0.75 ± 0.09	0.74 ± 0.09	0.78 ± 0.05	0.77 ± 0.09
BI-RADS 4C	0.76 ± 0.08	0.76 ± 0.09	0.79 ± 0.07	0.75 ± 0.07
BI-RADS 5	0.77 ± 0.06	0.76 ± 0.06	0.81 ± 0.02	0.77 ± 0.06
BI-RADS 6	0.73 ± 0.1	0.73 ± 0.1	0.69	0.75 ± 0.03
Volume of lesion				
0–5 cm3	0.77 ± 0.09	0.77 ± 0.1	0.79 ± 0.06	0.75 ± 0.07
5–20 cm3	0.74 ± 0.08	0.73 ± 0.08	0.74 ± 0.09	0.75 ± 0.09
20–100 cm3	0.72 ± 0.06	0.72 ± 0.06	0.81	0.68 ± 0.01

The reported values represent the mean DSCs across five folds after training with five-fold cross-validation. The ± values indicate the standard deviation of DSCs within each category. Categories without a ± value contain only one sample.

**Table 5:** HD obtained from five-fold cross-validation inference results across all datasets, categorized by BI-RADS levels and lesion volume.

	Total cohort (n = 292)	Training set (n = 204)	Validation set (n = 29)	Test set (n = 59)
All lesions	61.29 ± 36.58	62.86 ± 37.14	51.54 ± 35.66	60.67 ± 34.87
BI-RADS category				
BI-RADS 3	53.1 ± 37.27	60.81 ± 39.86	24.17 ± 12.58	46.99 ± 33.14
BI-RADS 4A	46.24 ± 41.94	43.42 ± 42.4	55.91 ± 49.86	52.01 ± 40.93
BI-RADS 4B	59.75 ± 35.45	62.77 ± 35.22	49.07 ± 34.33	56.67 ± 38.57
BI-RADS 4C	63.23 ± 34.16	63.84 ± 35.18	61.22 ± 39.6	61.83 ± 29.14
BI-RADS 5	78.66 ± 35.16	80.61 ± 35.14	49.58 ± 33.07	87.68 ± 32.84
BI-RADS 6	63.73 ± 36.11	70.38 ± 34.2	44.39	13.59 ± 1.08
Volume of lesion				
0–5 cm3	43.63 ± 31.82	45.06 ± 33.26	31.53 ± 22.95	45.09 ± 30.24
5–20 cm3	90.41 ± 24.88	91.07 ± 25.85	87.4 ± 21.42	89.62 ± 24.1
20–100 cm3	85.0 ± 18.38	83.93 ± 18.68	108.79	81.63 ± 15.45

The reported values represent the mean HD across five folds after training with five-fold cross-validation. The ± values indicate the standard deviation of HD within each category. Categories without a ± value contain only one sample.

**Fig. 6:**
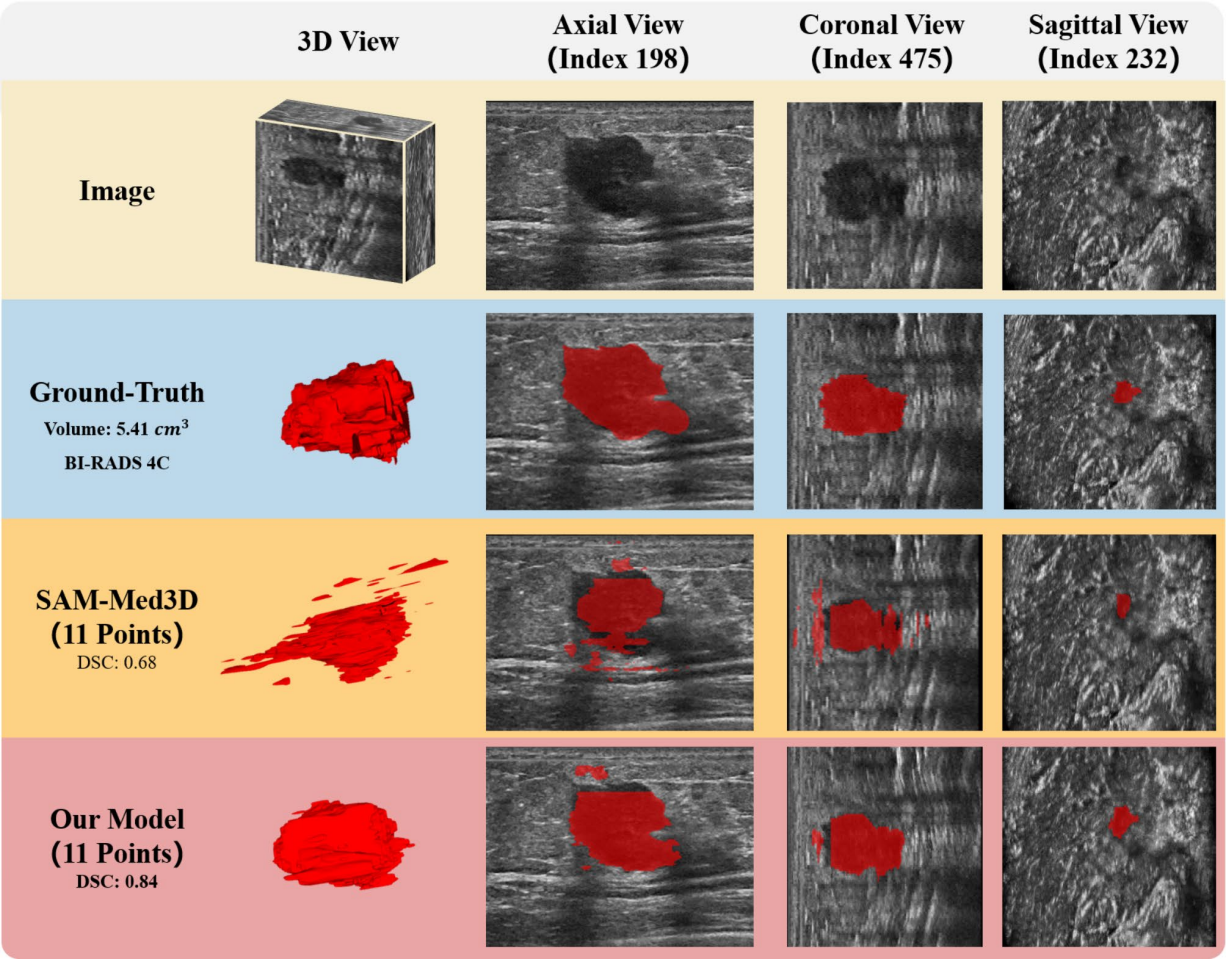
Illustration of ground truth, segment result of SAM-Med3D and SAM-Med3D with customized module.

Analysis revealed that the proposed model achieved good average inference results across different lesion characteristics. However, there were variations at the extremes of specific feature categories. From the perspective of BI-RADS grading characteristics, the poorest inference performance in the whole dataset was observed in the BI-RADS 6 category. The model’s best performance across BI-RADS categories reached approximately 0.9 DSC score. In terms of lesion volume size characteristics, the model’s overall average inference capability was relatively balanced. Among the 292 samples, there were three cases where the inference results had a DSC score of less than 0.5, with values of 0.37, 0.45, and 0.496, respectively. The poorest performance was observed in small volume samples (0–5 cm^3^) within the BI-RADS 4C category across all datasets. The best performance across different size categories ranged from 0.8 to 0.9 DSC score. Figure 6 illustrates a comparison between the segmentation results of our model, SAM-Med3D, and the ground truth.

Additionally, the SAM-Med3D model with the customized module was evaluated on 200 cases from the TDSC-ABUS 2023 challenge. The mean DSC score reached 0.786. For further comparative analysis of model performance, inference was conducted on the test set of the TDSC-ABUS 2023 challenge, achieving an average DSC score of 0.790. The top score on the challenge’s segmentation leaderboard was 0.729, indicating an improvement of 6.1%. To investigate the impact of dataset size on segmentation performance, we expanded the training dataset by incorporating the training set from the TDSC-ABUS 2023 dataset. The final experimental results demonstrate that a larger training dataset improved the segmentation performance, yielding a 0.01 increase in the mean Dice score on test set. This demonstrates that increasing the amount of training data can contribute to better segmentation performance. Furthermore, we conducted a comparative experiment between our method and a 3D version of the traditional fully automatic segmentation model, ResNet, without using five-fold cross-validation. The results indicate that the ResNet 3D model achieved only a DSC score of 0.44 on the test set.

## Discussion

This study found that the SAM-Med3D model with a customized module can be used for semi-automatic volumetric segmentation of breast tumor with variable accuracy, depending on the tumor’s shape and size. These results can serve as a preliminary judgment of the tumor area, size, and shape in a computer-aided diagnosis (CAD) system, reducing the workload for sonologists during the annotation process. The customized model can achieve a certain degree of correct segmentation for tumors of various sizes and BI-RADS categories, with an overall competitive segmentation ability. The segmentation results approximate those of professional annotators and can perform well on 3D ABUS images with different dimensions and spatial information from the test data, demonstrating good generalization performance.

In clinical diagnosis, the BI-RADS lexicon and assessment categories proposed by the American College of Radiology are often used to screen patients’ radiological examinations, reducing misunderstandings caused by inconsistent standards and increasingly complex diagnostic terms among different doctors^29^. However, studies have found that even under standard protocols, the diagnosis and screening of patients’ radiological examinations are largely limited by the examiner’s experience and workload, leading to a significant proportion of images being reclassified after initial evaluation^30^. A well-trained deep-learning model does not suffer from these constraints. In the SAM-Med3D model, for each image input and radiologist’s click on the lesion area, the model considers the entire image’s features to generate an initial breast cancer tumor region without changing the model or its customized weight parameters. Using a CAD system to assist in the preliminary generation of lesion areas can reduce the ambiguity in diagnosis caused by the time spent on each image.

ABUS, as an emerging breast radiology technology, can better detect structural distortions with coronal views and accurately measure cancerous tumors larger than 5 cm with its large probe coverage^31^. For women with high breast density, who have a higher risk of breast cancer, mammography is commonly recommended for potential breast cancer screening. However, studies have shown that mammography alone may miss occult tumors, whereas ultrasound can significantly improve detection rates^32^. However, both the interpretation and execution of ABUS examinations take longer compared to handheld ultrasound (HHUS)^33^. An AI model capable of accurately identifying and marking tumor areas can significantly reduce interpretation time, further enhancing ABUS as a breast cancer screening tool.

Semi-automatic segmentation in the medical field has seen increasing applications, such as using convolutional neural networks for semi-automatic liver cancer tumor segmentation in MRI images^34^, and a semi-automatic computer-aided interpolation method for fast and accurate segmentation of the intestines and stomach^35^. However, for large-scale segmentation tasks, semi-automatic segmentation remains time-consuming, whereas fully automatic segmentation can further leverage the advantages of AI models.

In the future, our goal is to further improve the performance of our customized 3D ABUS segmentation module and develop fully automatic segmentation models, eventually applying these segmentation algorithms in various clinical settings. For example, assisting doctors in screening potential breast cancer patients to optimize the screening process’s efficiency and alleviate radiologists’ workload. Future research will also combine other examination results and patient information for multimodal ABUS tumor characterization assessments, automatically generating detailed AI examination reports for further review by experienced doctors, thus creating a more comprehensive CAD system to reduce hospital visit pressure.

Statistical analysis (Fig. 4 and Table 2) reveals an imbalance in tumor size and BI-RADS distribution, with 61.6% of samples having a tumor size of 0–5 cm^3^. Studies have shown that breast cancer is a heterogeneous disease with significant heterogeneity between tumors and within tumors^36^. The uneven distribution of samples makes it challenging for the model to learn a weight parameter suitable for all tumors, resulting in some samples having poor segmentation performance. In the future, we will introduce more clinical case samples to form a more balanced dataset and further optimize the model structure or introduce better modules to make the algorithm results closer to radiologists’ segmentation results.

In conclusion, based on the LoRA fine-tuning method, the customized module allows the SAM-Med3D model to provide competitive semi-automatic segmentation performance for 3D ABUS breast tumors. Compared to fine-tuning all parameters of SAM-Med3D, adding a very small customized weight parameter module can adapt the model well to specific downstream tasks. This study demonstrates the feasibility of using a semi-automatic segmentation large model with a customized module to assist doctors in diagnosing 3D ABUS examinations.

## Data Availability

The processed datasets used and analysed during the current study available from the corresponding author (MM. Shuzheng Chen: dr.susan@163.com) on reasonable request.
